# Effects of 22 traditional anti-diabetic medicinal plants on DPP-IV enzyme activity and glucose homeostasis in high-fat fed obese diabetic rats

**DOI:** 10.1042/BSR20203824

**Published:** 2021-01-22

**Authors:** Prawej Ansari, Mary P. Hannon-Fletcher, Peter R. Flatt, Yasser H.A. Abdel-Wahab

**Affiliations:** School of Biomedical Sciences, Ulster University, Coleraine, BT52 1SA, Co. Londonderry, Northern Ireland, United Kingdom

**Keywords:** DPP-IV, glucagon-like peptide-1, hyperglycaemia, insulin, Medicinal plants, type 2 diabetes

## Abstract

The present study investigated the effects of hot water extracts of 22 medicinal plants used traditionally to treat diabetes on Dipeptidyl peptidase-IV (DPP-IV) activity both *in vitro* and *in vivo* in high-fat fed (HFF) obese-diabetic rats. Fluorometric assay was employed to determine the DPP-IV activity. For *in vivo* studies, HFF obese-diabetic rats were fasted for 6 h and blood was sampled at different times before and after the oral administration of the glucose alone (18 mmol/kg body weight) or with either of the four most active plant extracts (250 mg/5 ml/kg, body weight) or established DPP-IV inhibitors (10 μmol/5 ml/kg). DPP-IV inhibitors: sitagliptin, vildagliptin and diprotin A, decreased enzyme activity by a maximum of 95–99% (*P*<0.001). Among the 22 natural anti-diabetic plants tested, *Anogeissus* *Latifolia* exhibited the most significant (*P*<0.001) inhibitory activity (96 ± 1%) with IC_50_ and IC_25_ values of 754 and 590 μg/ml. Maximum inhibitory effects of other extracts: *Aegle marmelos, Mangifera indica, Chloropsis cochinchinensis, Trigonella foenum-graecum* and *Azadirachta indica* were (44 ±7%; 38 ± 4%; 31±1%; 28±2%; 27±2%, respectively). A maximum of 45% inhibition was observed with >25 μM concentrations of selected phytochemicals (rutin). *A.* *latifolia, A. marmelos, T. foenum-graecum* and *M. indica* extracts improved glucose tolerance, insulin release, reduced DPP-IV activity and increased circulating active GLP-1 in HFF obese-diabetic rats (*P*<0.05–0.001). These results suggest that ingestion of selected natural anti-diabetic plants, in particular *A. latifolia, A. marmelos, T. foenum-graecum* and *M. indica* can substantially inhibit DPP-IV and improve glucose homeostasis, thereby providing a useful therapeutic approach for the treatment of T2DM.

## Introduction

Diabetes Mellitus has become a worldwide concern, manifesting as one of the most major health issues within the world’s population. There are several forms of diabetes, including gestational diabetes, but Type 1 and Type 2 diabetes are by far the most prevalent. Type 2 diabetes (T2DM), most often associated with obesity, is a particularly widespread disease and many patients from all over the globe are afflicted by this condition. T2DM patients are characterized by impaired β-cell function and insulin secretion together with tissue insulin resistance [1]. Since the prevalence and associated complications of T2DM are so damaging, more effective therapies are being sought to either delay or prevent the progression of T2DM [2]. As so, plants are most reliable source as studies to date found that they contain series of potential phytogroups including alkaloids, glycosides, terpenoids, phenolic, flavonoids and plant-derived peptides, each of them has shown potential antidiabetic activity in different experiment [2]. Besides, the diet of patients with T2DM plays a vital role in helping to maintain blood glucose control involving such factors as energy density, carbohydrate content, dietary fiber and natural products that may directly or indirectly affect the absorption of nutrients or the secretion and action of insulin [3]. Hormones released by the gut in response to nutrient absorption, most notably GIP and GLP-1, also play an important role in modulating post-prandial hyperglycemia [4]. These hormones have a very short circulating half-life due to inactivation by the enzyme DPP-IV that cleaves the first two amino acids from the N-terminals producing GIP (3-42) and GLP-1 (9-36) [5]. This is why DPP-IV inhibitors are beneficial in the treatment of T2DM. Anti-diabetic drugs like metformin and nateglinide that respectively target insulin action and insulin secretion can, at high concentrations, also suppress DPP IV enzyme activity and such action may partly explain use of nateglinide as prandial insulin releasing agent that augments GLP-1 levels [6].

Having adequate bioactive insulin in the circulation is the key to control of glucose homeostasis as the hormone is unique in stimulating tissue glucose uptake and limiting hepatic glucose output. DPP-IV interferes with normal insulin action by degrading and therefore diminishing the insulinotropic and other β-cell actions of GLP-1 and GIP [4]. Natural resources are being explored to find new dietary ways to promote healthy blood glucose control including manipulation of the microbiome [7]. Over the years, many studies have revealed the anti-diabetic activity of plants used traditionally for the treatment of diabetes and defined their actions mediated via effects on the gastrointestinal processing of food and both the secretion and action of insulin [8–10]. More recently, dietary components including dairy, tuna, rice, salmon and amaranth have been found to exhibit DPP-IV inhibitory properties *in vitro* [11,12].

In the present work, 22 traditional medicinal plants with proven anti-diabetic activity were selected to assess their effects on DPP-IV enzyme activity *in vitro* (Tables 1 and 2). Furthermore, four of the most effective plants (*A. latifolia, A. marmelos, T. foenum-graecum* and *M. indica*) were selected to assess their acute effects on plasma DPP-IV activity, glucose-lowering and insulin-releasing properties in high fat fed obese-diabetic rats.

**Table 1:** Traditional use of selected medicinal plants treatment for diabetes

Plants	Traditional Uses	References
*Acacia catechu* (L.f.) Willd.	Diabetes, obesity, asthma, bronchitis, anaemia, diarrhoea	[34,35]
*Bunium persicum* (Boiss.) B.Fedtsch.	Obesity, gastrointestinal and urinary disorders, diarrhoea, asthma	[36]
*Eugenia jambolana* Lam.	Diabetes, cancer, enteric disorders, renal problems	[37,38]
*Linum usitatissimum* L.	Gastrointestinal disorders, asthma, bronchitis, pulmonary tuberculosis, gingival disorders, atherosclerosis	[39,40]
*Santalum album* L.	Inflammation, anti-septic, fever, carminative, diuretic, hypotensive, memory booster	[41]
*Selaginella bryopteris* (L.)	Jaundice, chronic tracheitis, lung cancer, venereal diseases, colitis, diuretic problems	[42,43]
*Sesamum indicum* (White)	Dietary fibre, joint inflammation, toothache, scrapes, cuts	[44,45]
*Tamarindus indica*	Inflammation, rheumatism, diarrhoea, dysentery, respiration conditions, malaria, gonorrhoea	[46]
*Terminalia arjuna* (Roxb. ex DC.)	Diabetes, cirrhosis, anaemia, cardiovascular disorders, viral diseases	[47,48]
*Azadirachta indica*	Diabetes, urinary and gastrointestinal problems, skin diseases, blood pressure and cholesterol	[49]
*Anogeissus latifolia* (Roxb. ex DC.)	Diabetes, haemorrhages, diarrhoea, dysentery, skin diseases, leprosy, hepatopathy	[50]
*Albizia lebbeck* (L.) Benth.	Respiratory disease, skin diseases, inflammation, diarrhoea, edema	[51,52]
*Cudrania cochinchinensis* (Lour.)	Gonorrhoea, rheumatism, jaundice, hepatitis, boils, scabies, bruising	[53]
*Cassia fistula* L.	Diabetes, jaundice, piles, rheumatism ulcers, skin eruptions, eczema, heart diseases, asthma, liver disorder	[54,55]
*Dalbergia sissoo* DC.	Bronchitis, inflammations, gonorrhoea, digestive disorders, colorectal cancer, bacterial infections	[56]
*Swertia chirayita* (Roxb.)	Diabetes, hypertension, liver disorders, malaria, hepatitis, inflammation, digestive diseases, epilepsy	[57,58]
*Withania coagulans* (Stocks)	Chronic degenerative diseases, diabetes	[59]
*Glycyrrhiza glabra* L.	Dyspepsia, belching, gas stomach ache, intestinal and liver colics, ulcerated wounds and gastritis	[60]
*Momordica charantia* L.	Diabetes, hypertension, obesity, cancer, hyperlipidaemia, digestive disorders, microbial infections	[61,62]
*Mangifera indica* L.	Diabetes, hypertension, anaemia, haemorrhage, asthma, gastric disorders	[63,64]
*Aegle marmelos* (L.) Corrêa	Diabetes, inflammations, asthma, ophthalmia, diarrhoea, dysentery, cardiac ailments	[65]
*Trigonella foenum-graecum* L.	Diabetes, hypercholesterolemia, edema lung congestion sinus, indigestion, baldness	[66,67]

**Table 2:** Antidiabetic actions of selected traditional plants treatment for diabetes

Plants	^1^Hyperglycemia	^2^Insulin secretion	^3^Glucose uptake and metabolism	References
*Acacia catechu*	↓	↑	ND	[68]
*Bunium persicum*	↓	ND	↑	[69]
*Eugenia jambolana*	↓	↑	↑	[70]
*Linum usitatissimum*	↓	↑	↑	[71,72]
*Santalum album*	↓	↑	↑	[73]
*Selaginella bryopteris*	↓	↑	↑	[74]
*Sesamum indicum* (White)	↓	ND	↑	[75]
*Tamarindus indica*	↓	↑	ND	[76]
*Terminalia arjuna*	↓	↑	↑	[77,78]
*Azadirachta indica*	↓	↑	↑	[79,80]
*Anogeissus latifolia*	↓	↑	ND	[81]
*Albizia lebbeck*	↓	↑	↑	[82,83]
*Chloropsis cochinchinensis*	↓	↑	↑	[84]
*Cassia fistula*	↓	↑	↑	[85,86]
*Dalbergia sisso*	↓	ND	ND	[87,88]
*Swertia chirrayita*	↓	↑	↑	[89]
*Withania coagulans*	↓	ND	ND	[90,91]
*Licorice glyceriza*	↓	↓	ND	[16,92,93]
*Momardica chirantia*	↓	↑	ND	[94,95]
*Mangifera indica*	↓	↑	↑	[96,97]
*Aegle marmelos*	↓	↑	↑	[21]
*Trigonella foenum graecum*	↓	↑	ND	[22,98]

Effects of plant: - ↑, increase; ↓, decrease (beneficial effect on hyperglycemia); ND, effect not determined

1 Effects on hyperglycemia were demonstrated in mice or rats given streptozotocin or alloxan or high fat diet to induce diabetes.

2 Effects on insulin secretion were demonstrated *in vitro* using pancreatic β-cells or *in vivo* using blood plasma of rats or mice. Beneficial actions *in vitro* were dose-dependent and did not affect cellular viability at low concentrations.

3 Effects on glucose uptake and metabolism were demonstrated *in vitro* using isolated mouse abdominal muscle.

## Materials and methods

### Plant materials and preparation of extract

Twenty-two plants used traditionally to treat diabetes were purchased to assess their ability to inhibit DPP-IV enzyme activity and improve glycemic control. The plants selected and their traditional and pharmacological actions are given in Tables 1 and 2. All plant materials were sourced in India where they are the native species. Confirmation of identity for the plants was made by a taxonomist Prof. F. A. Khan, Head of Department of Botany, Benazir Govt. Science & Commerce College, Bhopal, Barkatullah University, Madhya Pradesh, India where the plant specimens have been deposited in the herbarium. The accession numbers (voucher specimen numbers) for 22 traditional medicinal plants are listed in Table 3.

**Table 3:** List of confirmation of identity of 22 traditional medicinal plants with their herbarium numbers

Plants	Collected parts of plants	Voucher specimen numbers
*Acacia catechu* (L.f.) Willd.	Bark	1721
*Bunium persicum* (Boiss.) B.Fedtsch.	Seed	1844
*Eugenia jambolana* Lam.	Seed	1681
*Linum usitatissimum* L.	Seed	1531
*Santalum album* L.	Bark	1168
*Selaginella bryopteris* (L.)	Leaf	1135
*Sesamum indicum* (White)	Seed	1219
*Tamarindus indica*	Seed	866
*Terminalia arjuna* (Roxb. ex DC.)	Bark	535
*Azadirachta indica*	Seed	1610
*Anogeissus latifolia* (Roxb. ex DC.)	Bark	1734
*Albizia lebbeck* (L.) Benth.	Bark	1761
*Cudrania cochinchinensis* (Lour.)	Bark	1241
*Cassia fistula* L.	Stalk	1321
*Dalbergia sissoo* DC.	Bark	335
*Swertia chirayita* (Roxb.)	Bark	581
*Withania coagulans* (Stocks)	Fruit	1196
*Glycyrrhiza glabra* L.	Root	2212
*Momordica charantia* L.	Seed	2378
*Mangifera indica* L.	Seed	2391
*Aegle marmelos* (L.) Corrêa	Leaf	1733
*Trigonella foenum-graecum* L.	Seed	681

All plant components (Tables 1–3) were dried and grounded to obtain a fine powder. About 1 g of each dried powder was infused using 40 ml of boiled water. Aqueous extracts were chosen based on traditional use and prior studies of plants selected. The infusion was left for 15 min before being filtered through Whatman no. 1 filter paper. After that, the filtrates were dried under a vacuum (Savant Speedvac; New York, U.S.A.) to produce plant extract that was used to perform DPP-IV inhibitory experiments. For this purpose, the dried extract was dissolved in a 100 mM Tris-HCl buffer at an initial concentration of 5 mg/ml.

### Determination of DPP-IV inhibitory activity *in vitro*

A fluorometric method was used to determine the DPP-IV inhibitory activity of plant extracts based on that described previously [6,13]. For *in vitro* studies, a 100 mM Tris-HCl buffer was prepared and adjusted to pH 8.0 using 100 mM Tris-base. Reactions were performed in 96-well black-walled, clear-bottomed microplates (Premier Scientific Ltd, Belfast, U.K.) using 8 mU/ml of DPP-IV enzyme and 200 μM of fluorescent substrate (Gly-Pro-AMC) with or without plant extract, known DPP-IV inhibitor or selected phytochemicals. These included caffeine, catechin, epicatechin, gallic acid, isoquercitrin, quercetin and rutin as well as the small molecule anti-diabetic drug nateglinide. DPP-IV assay was based on liberation of AMC (7-amino-4-methyl-coumarin) from DPP-IV substrate, Gly-Pro-AMC. Changes in fluorescence due to cleavage of the molecule by DPP-IV were measured with an excitation and emission at 370 and 440 nm with 2.5 nm slit width using a FlexStation 3 (Molecular Devices, California, U.S.A.). The inhibition of DPP-IV activity was calculated as the percentage of inhibition by each plant extract at various concentrations. Neither the plant extracts nor plasma samples showed any loss of activity when stored for many months at 20°C. It was checked in control experiments that the extracts did not themselves cleave the substrate or interfere with fluorescence measurements at the concentrations employed.

### Animals

Forty male Sprague-Dawley rats (Envigo, Huntingdon, U.K., approximately 380–400 g) were fed a high-fat diet (45% fat, 20% protein and 35% carbohydrate; 26.15 kJ/g total energy percent; Special Diet Service, Essex, U.K.), *ad libitum* for 5–6 weeks to induce obesity and glucose intolerance. An additional 10 age-matched rats were maintained on standard rodent diet (30% protein, 10% fat, and 60% carbohydrate; 12.99 kJ/g total energy percent; Trouw Nutrition, Cheshire, U.K.). High fat fed rats exhibited increased body weight (398.7 ± 1.6 g versus 384.7 ± 1.8 g; *P*<0.01), impaired oral glucose tolerance and enhanced glucose-induced insulin responses, indicative of insulin resistance compared with the lean control rats fed normal diet (Figure 3). These animals also exhibited significantly elevated HbA1c levels (5.90 ± 0.07% versus 4.57 ± 0.05%; *P*<0.001), measured by the point-of-care A1CNow+ kit (PTS Diagnostic, Indiana), indicative of mild diabetes. The animals were housed individually in an air-conditioned room at 22 ± 2°C with a 12-h light/dark cycle. All animal experiments were conducted in accordance with U.K. Animals (Scientific Procedures) Act 1986 and EU Directive 2010/63EU. All necessary steps were taken to prevent any potential animal suffering. The animal studies were approved by local Ulster Animal Welfare and Ethical Review Body (AWERB) committee (01/10/2016), as well as being covered under a U.K. Home Office Animal project/personal licence numbers PIL450, PIL1822 and PPL 2804, approved on 06/05/2016. All animals were maintained under specific pathogen-free conditions and experiments were conducted in the Biomedical and Behavioral Research Unit (BBRU) at Ulster University, Coleraine, U.K. Blood was collected from the cut tip of the tail of conscious animals without need for anaesthesia. No animals were culled.

### Determination of the acute effects of plant extracts on DPP-IV activity *in vivo*

High fat-fed rats were used to study DPP-IV inhibitory activity of four medicinal plants (*A. latifolia, A. marmelos T. foenum-graecum* and *M. indica*) *in vivo.* Sitagliptin and vidagliptin were used in comparison as positive controls. Animals were fasted for approximately 6 h prior to experimentation. Blood samples were collected from the cut tip of the tail from conscious rats before and after oral administration of glucose with or without plant extract (250 mg/5 ml/kg) or an established DPP-IV inhibitor (10 μmol/5 ml/kg) at 0, 30, 60, 120, 180, 240, 360 and 480 min. Samples were collected in chilled fluoride/heparin-coated micro centrifuge tubes followed by centrifugation at 12000 rpm for 5 min. Plasma was stored at −20°C for measurement of insulin, DPP-IV activity and active GLP-1 (7-36). An Ascencia Contour glucose meter (Bayer, Newbury, U.K.) was used to measure blood glucose and insulin was determined by dextran-charcoal radioimmunoassay [14]. Active GLP-1 (7-36) was determined in the plasma samples collected at 60 min using a specific GLP-1 (Active) ELISA Kit (EGLP-35K, Merck Millipore, Dorset, U.K.).

### Statistical analysis

Statistical analysis tests were performed by using Graph Pad-Prism 5. The results are represented as mean ± SEM. Data were analyzed using by unpaired Student’s *t* test (nonparametric, with two-tailed *P* values) and one-way ANOVA with repeated measures was used and adjusted using Bonferroni correction. *P* value of < 0.05 was considered significant.

## Results

### *In vitro* DPP-IV Inhibitory effects of plant extracts

**Figure 1:**
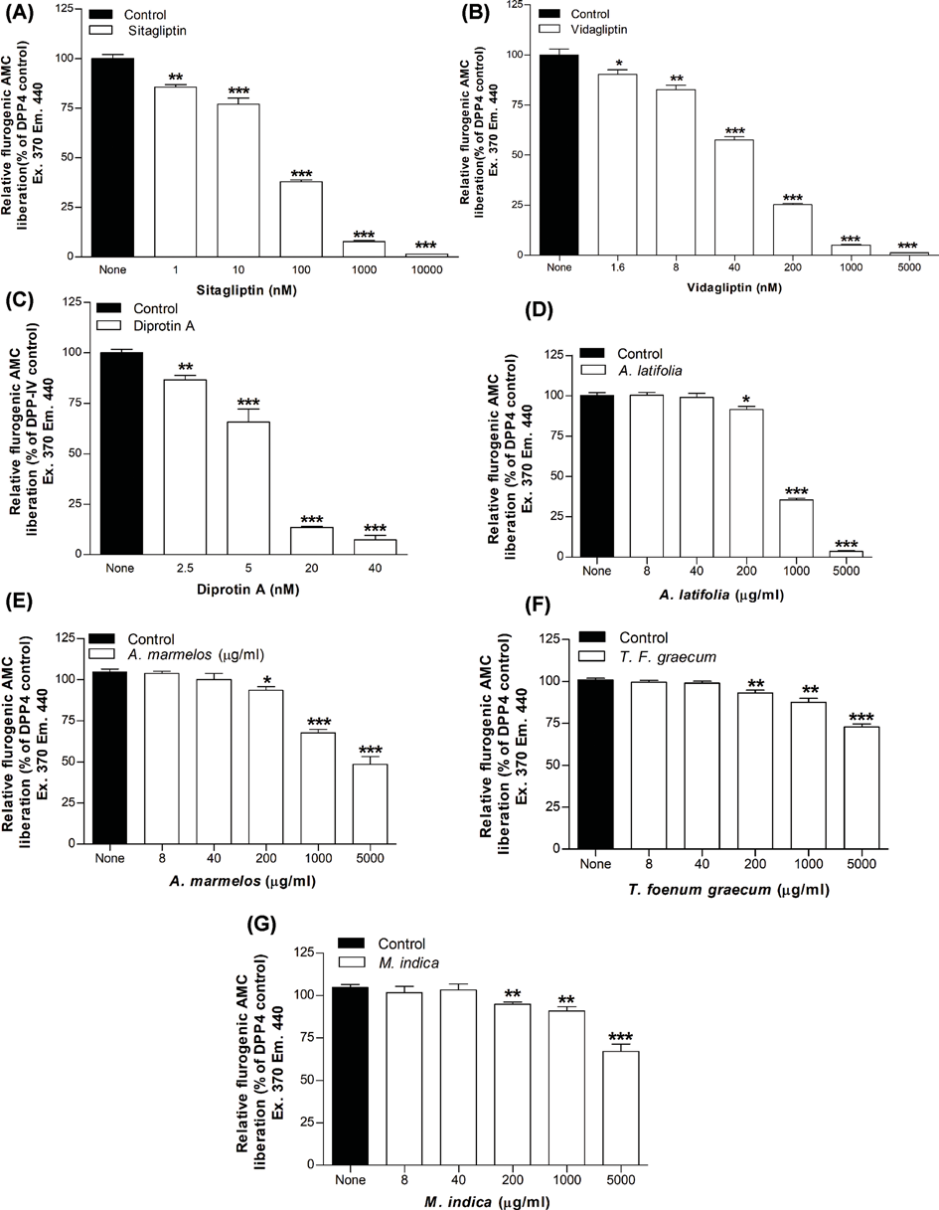
DPP-IV inhibitory activity of (A) Sitagliptin, (B) Vidagliptin, (C) Diprotin-A and hot water extract of four most potent plants (D) *A. latifolia*, (E) *A. marmelos*, (F) *T. foenum graecum* and (G) *M. indica* expressed as the bar chart (A–G) Values are mean ± SEM with *n*=4, *P<0.05, **P< 0.01 and ***P< 0.001, compared with control. Sitagliptin: 1–10,000 nM; Vidagliptin: 1.6–5000 nM and Diprotin A: 2.5–40 nM.

**Figure 2:**
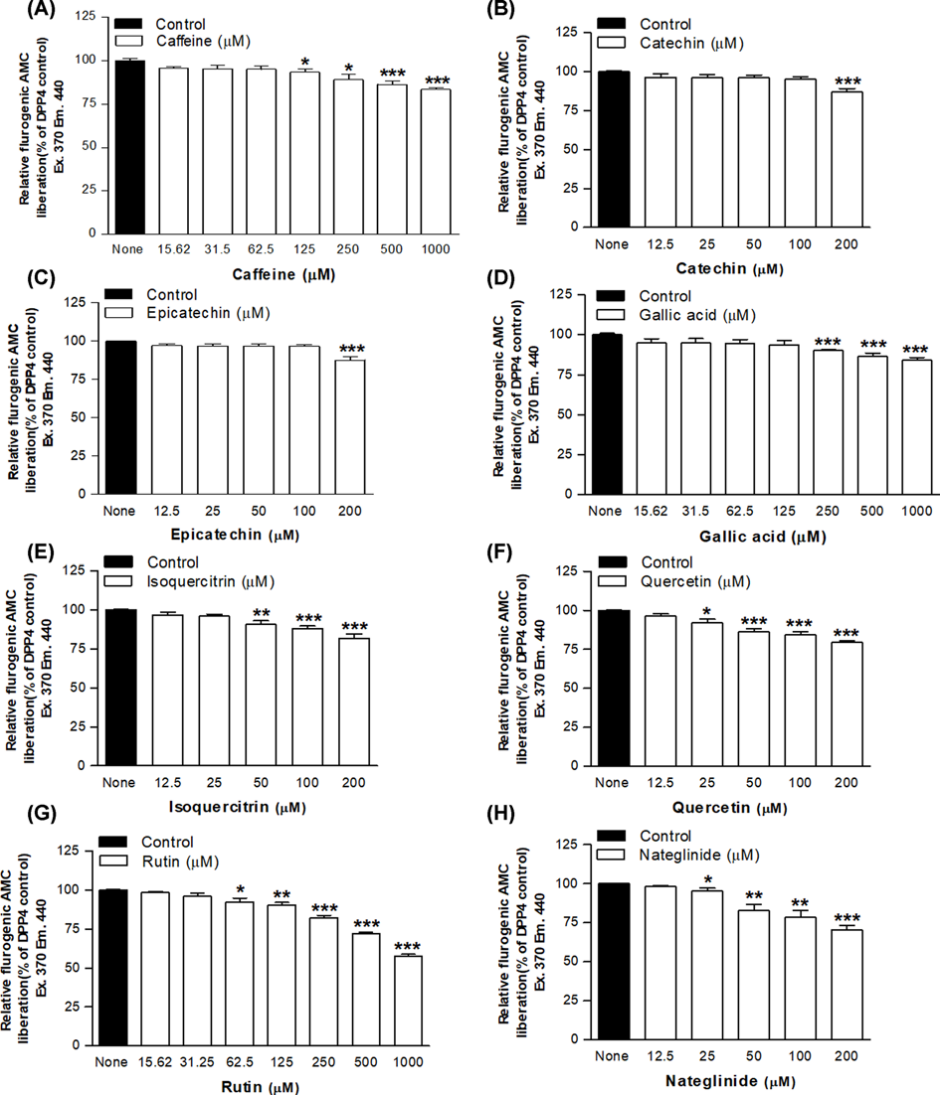
DPP-IV inhibitory activity of phytochemicals: (A) caffeine, (B) catechin, (C) epicatechin, (D) gallic acid, (E) isoquercitrin, (F) quercetin, (G) rutin and (H) the antidiabetic small molecule drug, nateglinide Values are mean ± SEM with *n*=4, *P<0.05, **P<0.01 and ***P< 0.001, compared with control.

The extracts from 22 different plants were evaluated *in vitro* to assess their effects on DPP-IV activity. Established DPP-IV inhibitors namely sitagliptin, vildagliptin and diprotin A were used as positive controls (Table 4 and Figure 1A–C). These inhibitors decreased DPP-IV enzyme activity by up to 99 ± 2.0%, 99 ± 3% and 95 ± 3%, with IC_50_ values of 2.04 × 10^−2^, 1.70 × 10^−2^ and 2.39 × 10^−3^ μg/ml (*P*<0.05–0.001, respectively (Table 4 and Figure 1A–C). In the presence of *A. latifolia* (bark), enzymatic AMC liberation from Gly-Pro-AMC was inhibited by 20 ± 1% to 96 ± 1% (*P*<0.05–0.001, Table 4, Figure 1D) at concentrations of 200–5000 μg/ml when compared with control. Moreover, *A. marmelos* (leaves), *M. indica* (seeds) and *T. foenum-graecum* (seeds) significantly inhibited DPP-IV enzyme activity at concentrations ranging from 200 to 5000 μg/ml (*P*<0.05–0.001, respectively, Table 4 and Figure 1E–G. The highest inhibitory effects of plant extracts were observed at 5000 μg/ml (44 ± 7%, 38 ± 4%, 31 ± 1% and 28 ± 2%, *P*<0.001, respectively, Table 4) as compared with control. The other plant extracts were found to inhibit DPP-IV activity in between 9 ± 1% and 27 ± 2% (*P*<0.05–0.001, Table 4) when tested at a concentration of 5000 μg/ml. The phytochemicals responsible for the inhibitory action are unknown but several possible candidates known to be present in the plant collection were tested. These included caffeine, catechin, epicatechin, gallic acid, isoquercitrin, quercetin and rutin. As shown in Figure 2A–G, each inhibited DPP-IV with the majority exhibiting lower effective concentrations of 125–200 μM. Isoquercitrin and quercetin inhibited at 25–50 μM whereas rutin was particularly effective with maximal inhibition of 45% and IC_25_ value of 306 μM. The effect was similar to the established insulinotropic drug nateglinide (Figure 2H).

**Table 4:** DPP-IV inhibitory activity of established inhibitors and hot water extract of various traditional plants

Plants/ Inhibitors	Lower effective concentration (μg/ml)	Maximum inhibitory effect (%)	IC_25_ (μg/ml)	Estimated IC_50_ (μg/ml)
Sitagliptin (Inhibitor)	4.07 × 10^−4^	99 ± 2‡	2.04 × 10^−3^	2.04 × 10^−2^
Vidagliptin (Inhibitor)	6.07 × 10^−4^	99 ± 3‡	2.43 × 10^−3^	1.70 × 10^−2^
Diprotin A (Inhibitor)		95 ± 3‡	1.02 × 10^−3^	2.39 × 10^−3^
*Anogeissus latifolia*	200	96 ± 1‡	590	754
*Aegle marmelos* (L.) Corrêa	200	44 ± 7†	446	790
*Mangifera indica* L.	200	38 ± 4‡	2000	—
*Cudrania cochinchinensis* (Lour.)	1000	31 ± 1‡	4,050	—
*Trigonella foenum-graecum* L.	200	28 ± 2‡	4,700	—
*Azadirachta indica*	1000	27 ± 2‡	4,720	—
*Tamarindus indica*	200	23 ± 2‡	—	—
*Terminalia arjuna* (Roxb. ex DC.)	200	22 ± 1‡	—	—
*Acacia catechu* (L.f.) Willd.	5000	22 ± 7*	—	—
*Withania coagulans* (Stocks)	1000	17 ± 3†	—	—
*Sesamum indicum* (White)	40	19 ± 5*	—	—
*Albizia lebbeck* (L.) Benth.	5000	19 ± 4†	—	—
*Cassia fistula* L.	5000	19 ± 6*	—	—
*Santalum album* L.	40	17 ± 5*	—	—
*Eugenia jambolana* Lam.	40	14 ± 5*	—	—
*Selaginella bryopteris* (L.)	1000	13 ± 4*	—	—
*Momordica charantia* L.	1000	13 ± 2†	—	—
*Swertia chirayita* (Roxb.)	5000	12 ± 1†	—	—
*Glycyrrhiza glabra* L.	200	12 ± 2*	—	—
*Bunium persicum* (Boiss.)	200	11 ± 3*	—	—
*Dalbergia sissoo* DC.	5000	10 ± 2*	—	—
*Linum usitatissimum* L.	40	9 ± 1*	—	—

DPP-IV inhibitory activity of hot water extracts of various plants when incubated with Gly-Pro-AMC (200 μM) plus DPP4 (8 mU/ml^−1^) for 30 min at 37°C. Sitagliptin, Vidagliptin and Diprotin A were used as established inhibitors. Values are mean ± SEM with *n*=4. *P<0.05, †*P*<0.01 and ‡*P*<0.001 compared with control group Gly-Pro-AMC (200 μM) + DPP4 (8 mU/ml^−1^) alone. The calculated IC_50_ (μg/ml) was an estimate.

### Acute effects of plants extract on glucose tolerance and plasma insulin in high fat-fed rats

**Figure 3:**
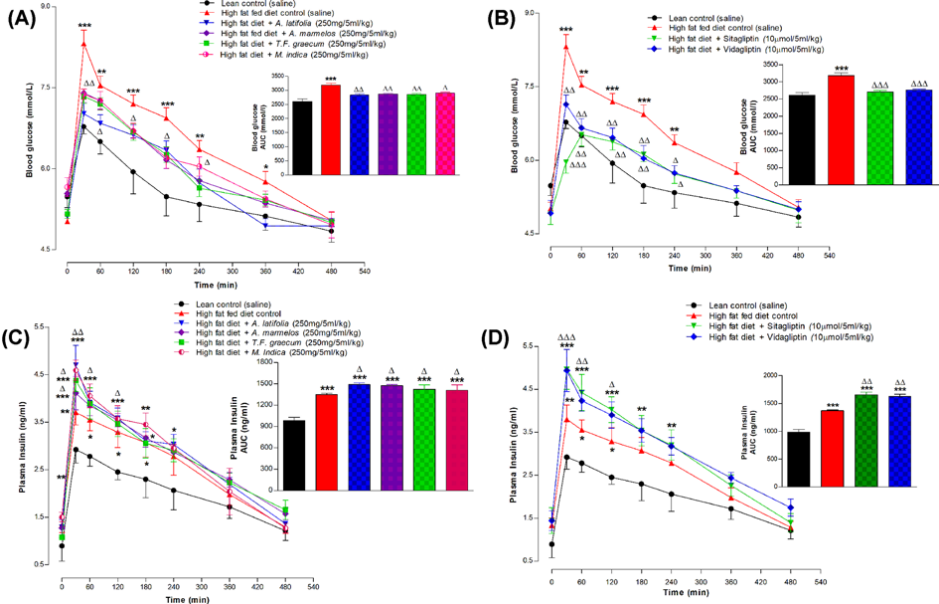
Acute effects of hot water extract of four most potent plants (A and C) *A. latifolia, A. marmelos, T. foenum* graecum, *M. indica* and (B and D) DPP-IV inhibitors: sitagliptin and vidagliptin on (A and B) glucose tolerance and (C and D) plasma insulin in high-fat fed rats expressed as line graphs and area under the curve Blood glucose and plasma insulin were measured prior to and after oral administration of glucose alone (18 mmol/kg body weight, control) or in combination with plant extract (250 mg/5 ml/kg body weight,), Sitagliptin or Vidagliptin (both at 10 μmol/5 ml/kg, body weight). Values are mean ± SEM with *n*=6, *P<0.05, **P<0.01 and ***P<0.001, compared with lean rats and ^Δ^*P*<0.05, ^ΔΔ^*P*<0.01 and ^ΔΔΔ^*P*<0.001 compared with high-fat fed controls.

Four plants (*A. latifolia, A. marmelos, T. foenum-graecum* and *M. indica*), the most potent in inhibiting *in vitro* DPP IV enzyme activity, were selected for evaluation of effects on DPP IV activity and oral glucose tolerance in high-fat fed rats. Hot water extracts (250 mg/5 ml/kg) substantially improved the glycemic excursion from 30 to 240 min and increased plasma insulin from 30 to 120 min as compared with oral administration of glucose alone (*P*<0.05–0.001; Figure 3A,C). Established DPP-IV inhibitors (sitagliptin and vildagliptin) also improved glucose tolerance and insulin release following oral administration (*P*<0.05–0.001; Figure 3B,D).

### Acute effects of plant extracts on circulating DPP-IV activity and active GLP-1 (7-36) in high fat-fed rats

**Figure 4:**
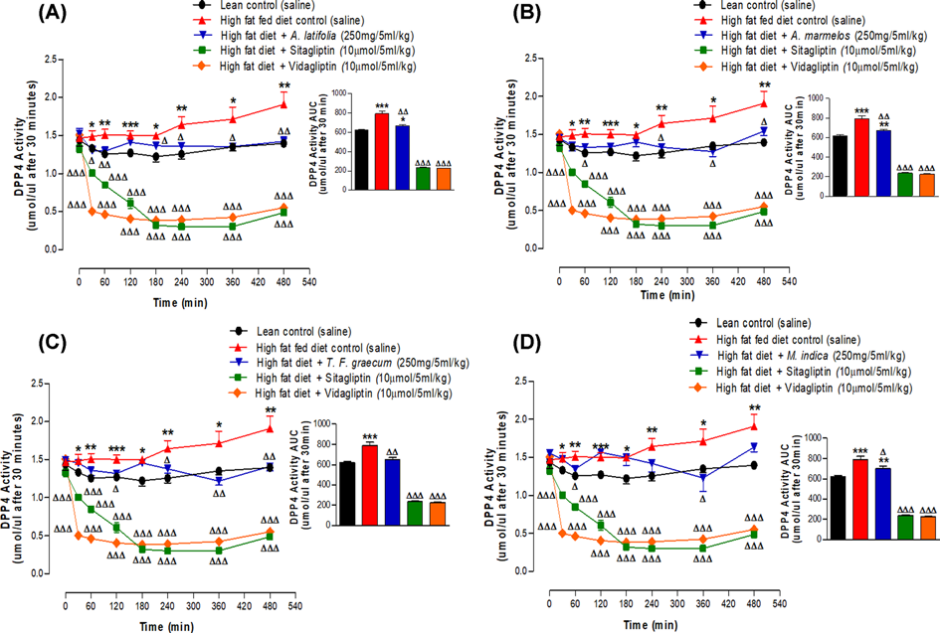
Acute effects of hot water extract of four most potent plants (A) *A. latifolia*, (B) *A. marmelos*, (C) *T. foenum graecum* and (D) *M. indica* on DPP-IV activity in high-fat fed rats expressed as line graphs and area under the curve Plasma DPP-IV activity was measured prior to and after oral administration of glucose alone (18 mmol/kg body weight, control) or in combination with plant extract (250 mg/5 ml/kg body weight), Sitagliptin or Vidagliptin (each at 10 μmol/5 ml/kg body weight). DPP-IV activity was determined by Gly-Pro-AMC (200 μM) cleavage. Values are mean ± SEM with *n*=6, *P<0.05, **P<0.01 and ***P<0.001, compared with lean rats and ^Δ^*P*<0.05, ^ΔΔ^*P*<0.01 and ^ΔΔΔ^*P*<0.001 compared with high-fat fed controls.

**Figure 5:**
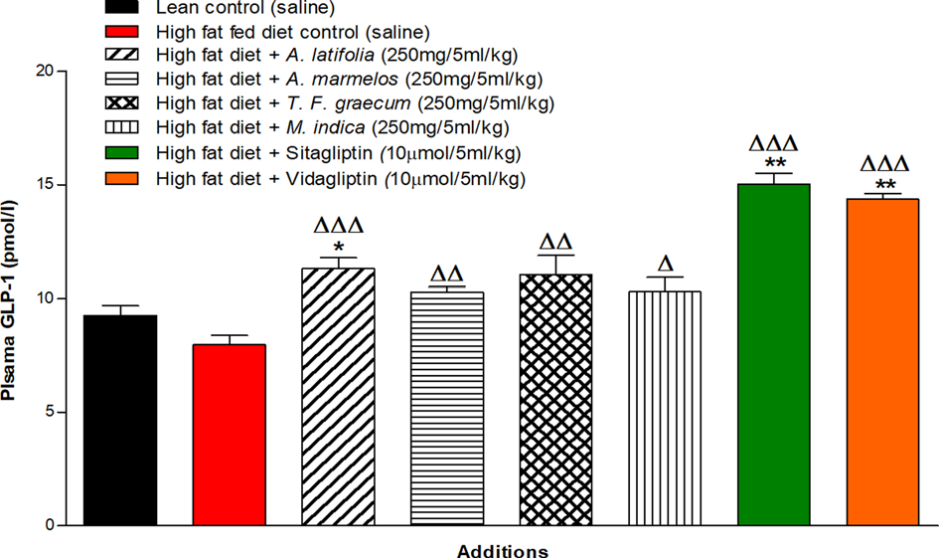
Acute effects of hot water extract of four most potent plants: *A. latifolia, A. marmelos, T. foenum graecum* and *M. indica* on plasma active GLP-1 (7-36) in high-fat fed rats Plasma active GLP-1 (7-36) concentrations was measured at 60 min after oral administration of glucose alone (18 mmol/kg body weight, control) or in combination with plant extract (250 mg/5 ml/kg body weight), Sitagliptin or Vidagliptin (both at 10 μmol/5 ml/kg, body weight). Values are mean ± SEM with *n*=6, *P<0.05, **P<0.01 compared with lean rats and ^Δ^*P*<0.05, ^ΔΔ^*P*<0.01 and ^ΔΔΔ^*P*<0.001 compared with high-fat fed controls.

Sitagliptin and vildagliptin significantly reduced DPP-IV activity in high-fat fed rats (*P*<0.001, Figure 4A–D). Hot water extracts (250 mg/5 ml/kg) of *A. latifolia, A. marmelos, T. foenum-graecum* and *M. indica* also significantly decreased *in vivo* DPP-IV enzyme activity compared with glucose alone (*P*<0.05–0.01, Figure 4A–D). The effects of *A. latifolia* and *T. foenum-graecum* were particularly prominent with a sustained inhibition of DPP-IV activity from 30 min onwards (*P*<0.05–0.01, Figure 4A,C). Lesser but still significant effects were observed with *A. marmelos* and *M. indica* extracts (*P*<0.05, Figure 4B,D). As shown in Figure 5, active GLP-1 (7-36) concentrations in plasma were significantly increased by 32–45% (*P*<0.05–0.01) at 60 min after administration of each plant extract. An 81–89% increase was observed with sitagliptin and vidagliptin (*P*<0.001; Figure 5).

## Discussion

DPP-IV inhibitors are used in the treatment of T2DM based on their ability to extend postprandial levels of circulating plasma GLP-1 and GIP, thereby improving insulin secretion and helping to maintain good blood glucose control. Since their introduction to the clinic [15], this drug class has proven to be effective and highly popular. Weight reduction and glycaemic control are inferior to the related family of GLP-1 mimetics but DPP-IV inhibitors have the advantage of being orally active, thereby avoiding the need for daily injections and increasing patient compliance. In certain developing countries, the limited availability and cost of these and other modern medicines, such as metformin, sulphonylureas, thiazolidenediones, SGLT2 inhibitors and insulin formulations, have resulted in increasing attention being paid to traditional plant medicines with reputed anti-diabetic activity for treatment of T2DM [3,8,10,16–18].

A considerable number of plants have been used traditionally for the treatment for diabetes and its complications but only a limited number have been subjected to scientific scrutiny and fewer still scrutinized for the mechanisms responsible for their anti-diabetic effects [9,16,19]. In the present study, we have examined 22 medicinal plants with proven glucose-lowering ability (Table 2) to assess whether part of their mode of action relates to an ability to inhibit DPP-IV. Hot water extracts of every plant studied exhibited some degree of DPP-IV inhibition *in vitro* ranging from 9 to 96%, but the most substantial effects were observed (in descending order) with *A. latifolia* (bark), *A. marmelos* (leaves), *M. indica* (seeds), *T. foenum-graecum* (seeds), *C. cochinchinensis* (bark), and *A indica* (seeds). These plants inhibited DPP-IV by 27–96% with IC_25_ values of 446–4720 μg/ml. Although considerably less effective than pure preparations of sitagliptin and vildagliptin, these observations suggest that a component of the anti-diabetic actions of these plants may be due to inhibition of DPP-IV. This adds to the results of previous studies which have highlighted gastrointestinal effects of anti-diabetic plants and their ability to enhance insulin secretion and/or action [20–23].

Based on these *in vitro* results, the four most active plants were selected from the 22 initially screened for *in vivo* evaluation of effects on oral glucose tolerance, insulin secretion and plasma DPP-IV activity using high-fat fed rats. This included *A. latifolia, A. marmelos, T. foenum-graecum* and *M. indica.* The first two were particularly effective in inhibiting DPP-IV *in vitro* with up to 44-96% inhibition and IC_50_ values of 754-790 μg/ml. This compares with almost total inhibition of DPP-IV by sitagliptin and vildagliptin with IC_25_ and IC_50_ values of 2.04 × 10^−3^ to 2.43 × 10^−3^ μg/ml and 2.04 × 10^−2^ to 1.70×10^−2^ μg/ml, respectively. As expected, administration of either sitagliptin or vildagliptin orally to high fat fed obese-diabetic rats, together with glucose, induced a remarkable improvement in glucose tolerance and glucose-stimulated insulin secretion. This was associated with a 70–72% decrease in plasma DPP-IV activity known to result in strong augmentation of the stimulatory insulin-releasing effects of the incretin hormones GLP-1 and GIP. Consistent with this, circulating concentrations of active GLP-1 (7-36) were increased by 81–89% at 60 min following administration of these DPP-IV inhibitors. Extracts of *A. latifolia, A. marmelos, T. foenum-graecum* and *M. indica* also significantly inhibited DPP-IV enzyme activity and increased active GLP-1 (7-36) but by lesser extents of 12–18% and 32–45%, respectively. Interestingly, the glucose lowering actions of the four plant extracts were very similar to the DPP-IV inhibitors despite a smaller plasma insulin response. This indicates that other factors such as a delayed glucose absorption make a major contribution to the acute anti-hyperglycaemic activity of these plants *in vivo* [9,23]. Further long-term studies are required to determine how their effects compare with other plant-derived substances that exhibit anti-diabetic properties, such as metformin.

These results suggest that many plants used traditionally to treat diabetes have orally available constituents that inhibit DPP-IV, thereby contributing to their spectrum of actions which in the case of some might be significant [24,25]. Indeed, additional *in vitro* and *in vivo* studies on individual plant extracts have reported that other plant species inhibit DPP-IV activity [26,27]. These observations suggest that these plant extracts exert part of their insulinotropic effects via inhibition of DPP-IV with resultant preservation of active forms of GLP-1 (7-36) and GIP (1-42) released from intestinal enteroendocrine cells by feeding. Overall, our results together with these previous studies indicate that net effects on insulin secreting cells reflect combination of direct actions of glucose and other nutrients compounded by potentiating effects of incretin hormones that are favored by the concurrent inhibition of DPP-IV. Further extensive studies will be required to measure active and total forms of GLP-1 and GIP following administration of the plants studied but the few reports in the literature suggest that some plants with reputed anti-diabetic activity increase circulating GLP-1 [28]. The extent to which this may reflect enhanced secretion as opposed to decreased degradation by DPP-IV is unknown.

Although the present study describes DPP-IV lowering activity of many medicinal plants with anti-diabetic actions, few precise details are known about the nature of the phytochemicals that are absorbed and subsequently inhibit DPP-IV. All 22 plants examined inhibited DPP-IV activity *in vitro* to some extent suggesting that such chemicals are commonly encountered in the plant kingdom. Studies to date suggest that these include alkaloids, glycosides, terpenoids, phenolic, flavonoids as well as protein hydrolysates and peptides [27,2]. These may act at the molecular level by binding to the active site of the enzyme, thereby inhibiting interactions with natural substrates. Small peptides may also serve as competitive substrates as is the case with diprotin-A. Indeed, we demonstrated DPP-IV inhibitory action for caffeine, catechin, epicatechin, gallic acid, isoquercitrin, quercetin and rutin which may realistically contribute to the observed effects as they have been reported to be present in plants at levels of up to 2–30% by weight, not accounting for losses during our extraction procedure [29]. Rutin is the most abundant of these phytochemicals and was also shown to be the most effective inhibitor of DPP-IV, with an action broadly similar to that observed with the established anti-diabetic drug, nateglinide. The active plant constituents might, like this meglitinide, serve as effective prandial blood glucose regulators stemming partly from their ability to preserve active forms of GLP-1 and GIP released by feeding [6].

In the light of the DPP-IV inhibitory actions of the 22 plants tested plus the selected phytochemicals, it is notable that flavonoids and their metabolites have been reported to exhibit anti-diabetic activities. An inverse relationship has been suggested also between flavonoid intake and T2DM risk [30]. Phytochemicals other than those tested, such as anthocyanin, aspalathin, chrysin, eriodictyol, hispidulin, kaempferol, lepidoside, mangiferin, naringenin, naringin, procyanidin, rhamnoside, terpenoids, vitexin and *Lens culinaris* extracts have been shown also to exhibit DPP-IV inhibitory activity [27,31]. In addition, a number of other plants (such as *A. catechu, M. indica* and *A. marmelos*) have been reported to contain potential phenolic compounds that exert antioxidant effects and DPP-IV inhibitory activity [32].

## Conclusions

In conclusion, these findings indicate that a substantial proportion of plants used traditionally for the treatment of diabetes exhibit DPP-IV inhibitory activity which may contribute to their multiple glucose lowering actions. Such medicinal plants could provide an accessible therapy for diabetes particularly in populations without easy access to the recognized drugs. More work is required for isolation, identification and characterization of agents responsible for inhibition of DPP-IV but there is a good chance that multiple phytochemicals are involved in mediating such effects [33].

## Data Availability

All data are included in the manuscripts and the identified participant information (PA) is included in the Author Contribution section for the data collections.

## Open Access

Open access for this article was enabled by the participation of Ulster University in an all-inclusive *Read & Publish* pilot with Portland Press and the Biochemical Society under a transformative agreement with JISC.

## Institutional Animal Care

All animal experiments were conducted in accordance to U.K. Animals (Scientific Procedures) Act 1986 and EU Directive 2010/63EU. All necessary steps were taken to prevent any potential animal suffering. The animal studies were approved by local Ulster Animal Welfare and Ethical Review Body (AWERB) committee (01/10/2016), as well as being covered under a U.K. Home Office Animal project/personal licence numbers PIL450, PIL1822 and PPL 2804, approved on 06/05/2016. All animals were maintained under specific pathogen-free conditions, and all experiments were conducted in the Biomedical and Behavioral Research Unit (BBRU) at Ulster University, Coleraine, U.K. Blood samples were collected from the cut tip of the tail of conscious animals and they are not scarified.

## Abbreviations

AMC: 7-amino-4-methyl-coumarin
DPP-IV: dipeptidyl peptidase-IV
GIP: glucose-dependent insulinotropic polypeptide
GLP-1: glucagon-like peptide-1
Gly-Pro-AMC: Gly-Pro-7-Amino-4-Methyl-Coumarin
HFF: high-fat fed
IC_25_: 25 percent inhibitory concentration
IC_50_: 50 percent inhibitory concentration
OGTT: oral glucose tolerance test
T2DM: Type 2 diabetes mellites

## References

[1] Wang C.Y. and Liao J.K. (2013) A Mouse Model of Diet-Induced Obesity and Insulin Resistance. Methods Mol. Biol. 821, 421–433 10.1007/978-1-61779-430-8_27 PMC3807094 22125082

[2] Patil S.P., Goswami A., Kalia K. and Kate A.S. (2020) Plant-Derived Bioactive Peptides: A Treatment to Cure Diabetes. Int. J. Pept. Res. Ther. 26, 955–968 10.1007/s10989-019-09899-z 32435169 PMC7223764

[3] Gray A.M. and Flatt P.R. (1997) Nature's own pharmacy: The diabetes perspective. Proc. Nutr. Soc. 56, 507–517 10.1079/PNS19970051 9168558

[4] Holst J.J. (2019) From the Incretin Concept and the Discovery of GLP-1 to Today's Diabetes Therapy. Front. Endocrinol. 10, 10 10.3389/fendo.2019.00260 PMC6497767 31080438

[5] Deacon C.F. (2019) Physiology and Pharmacology of DPP-4 in Glucose Homeostasis and the Treatment of Type 2 Diabetes. Front. Endocrinol. 10, 80–80 10.3389/fendo.2019.00080 PMC6384237 30828317

[6] Duffy N.A., Green B.D., Irwin N., Gault V.A., McKillop A.M., O'Harte F.P. et al. (2007) Effects of antidiabetic drugs on dipeptidyl peptidase IV activity: nateglinide is an inhibitor of DPP IV and augments the antidiabetic activity of glucagon-like peptide-1. Eur. J. Pharmacol. 568, 278–286 10.1016/j.ejphar.2007.05.010 17573070

[7] Gérard C. and Vidal H. (2019) Impact of Gut Microbiota on Host Glycemic Control. Front. Endocrinol. 10, 1–29 10.3389/fendo.2019.00029 PMC6363653 30761090

[8] Swanston-Flatt S.K., Day C., Bailey C.J. and Flatt P.R. (1990) Traditional plant treatments for diabetes. Studies in normal and streptozotocin diabetic mice. Diabetologia 33, 462–464 10.1007/BF00405106 2210118

[9] Gray A.M. and Flatt P.R. (1999) Insulin-releasing and insulin-like activity of the traditional anti-diabetic plant Coriandrum sativum (coriander). Br. J. Nutr. 81, 203–209 10.1017/S0007114599000392 10434846

[10] Gawli K., Lakshmidevi N., Murthy S., Prakash H.S. and Niranjana S.R. (2011) Diabetes and medicinal plants-A review. Int. J. Pharm. Biomed. Sci. 2, 65–80

[11] Patil P., Mandal S., Tomar S.K. and Anand S. (2015) Food protein-derived bioactive peptides in management of type 2 diabetes. Eur. J. Nutr. 54, 863–880 10.1007/s00394-015-0974-2 26154777

[12] Liu R., Cheng J. and Wu H. (2019) Discovery of Food-Derived Dipeptidyl Peptidase IV Inhibitory Peptides: A Review. Int. J. Mol. Sci. 20, 463 10.3390/ijms20030463 PMC6387223 30678216

[13] McKillop A.M., Duffy N.A., Lindsay J.R., Green B.D., Patterson S., O'Harte F.P. et al. (2009) Insulinotropic actions of nateglinide in type 2 diabetic patients and effects on dipeptidyl peptidase-IV activity and glucose-dependent insulinotropic polypeptide degradation. Eur. J. Endocrinol. 161, 877–885 10.1530/EJE-09-0547 19755410

[14] Bailey C.J. and Flatt P.R. (1982) Hormonal control of glucose homeostasis during development and ageing in mice. Metabol. Clin. Exp. 31, 238–246 10.1016/0026-0495(82)90059-2 7043171

[15] Green B.D., Flatt P.R. and Bailey C.J. (2006) Dipeptidyl peptidase IV (DPP IV) inhibitors: A newly emerging drug class for the treatment of type 2 diabetes. Diabetes Vas. Dis. Res. 3, 159–165 10.3132/dvdr.2006.024 17160910

[16] Swanston-Flatt S.K., Flatt P.R., Day C. and Bailey C.J. (1991) Traditional dietary adjuncts for the treatment of diabetes mellitus. Proc. Nutr. Soc. 50, 641–651 10.1079/PNS19910077 1809971

[17] Swanston-Flatt S., Day C., Flatt P., Gould B. and Bailey C. (1989) Glycaemic effects of traditional European plant treatments for diabetes. Studies in normal and streptozotocin diabetic mice. Diab. Res. 10, 69–73 2743711

[18] Swanston-Flatt S.K., Day C., Bailey C.J. and Flatt P.R. (1989) Evaluation of traditional plant treatments for diabetes: Studies in streptozotocin diabetic mice. Acta Diabetol. Lat 26, 51–55 10.1007/BF02581196 2750445

[19] Naveen J. and Baskaran V. (2018) Antidiabetic plant-derived nutraceuticals: a critical review. Eur. J. Nutr. 57, 1275–1299 10.1007/s00394-017-1552-6 29022103

[20] Rios J.L., Andujar I., Schinella G.R. and Francini F. (2019) Modulation of Diabetes by Natural Products and Medicinal Plants via Incretins. Planta Med. 85, 825–839 31064029 10.1055/a-0897-7492

[21] Ansari P., Afroz N., Jalil S., Azad S.B., Mustakim M.G., Anwar S. et al. (2017) Anti-hyperglycemic activity of *Aegle marmelos* (L.) corr. is partly mediated by increased insulin secretion, alpha-amylase inhibition, and retardation of glucose absorption. J. Ped. Endocrinol. Metabol. 30, 37–47 10.1515/jpem-2016-0160 28002030

[22] Hannan J.M., Ali L., Rokeya B., Khaleque J., Akhter M., Flatt P.R. et al. (2007) Soluble dietary fibre fraction of *Trigonella foenum-graecum* (fenugreek) seed improves glucose homeostasis in animal models of type 1 and type 2 diabetes by delaying carbohydrate digestion and absorption, and enhancing insulin action. British J. Nutr. 97, 514–521 10.1017/S0007114507657869 17313713

[23] Gallagher A.M., Flatt P.R., Duffy G. and Abdel-Wahab Y.H.A. (2003) The effects of traditional antidiabetic plants on in vitro glucose diffusion. Nutr. Res. 23, 413–424 10.1016/S0271-5317(02)00533-X

[24] Kehinde B.A. and Sharma P. (2018) Recently isolated antidiabetic hydrolysates and peptides from multiple food sources: a review. Crit. Rev. Food Sci. Nutr. 60, 1–19 30463420 10.1080/10408398.2018.1528206

[25] Turdu G., Gao H., Jiang Y. and Kabas M. (2018) Plant dipeptidyl peptidase-IV inhibitors as antidiabetic agents: a brief review. Future Med. Chem. 10, 1229–1239 10.4155/fmc-2017-0235 29749760

[26] Bansal P., Paul P., Mudgal J., Nayak P.G., Pannakal S.T., Priyadarsini K.I. et al. (2012) Antidiabetic, antihyperlipidemic and antioxidant effects of the flavonoid rich fraction of *Pilea microphylla* (L.) in high fat diet/streptozotocin-induced diabetes in mice. Exp. Toxicol. Pathol. 64, 651–658 10.1016/j.etp.2010.12.009 21208790

[27] Lin S.R., Chang C.H., Tsai M.J., Cheng H., Chen J.C., Leong M.K. et al. (2019) The perceptions of natural compounds against dipeptidyl peptidase 4 in diabetes: from *in silico* to *in vivo*. Ther. Adv. Chronic Dis. 10, 1–16 10.1177/2040622319875305 PMC6753520 31555430

[28] Kim K.-S. and Jang H.-J. (2015) Medicinal Plants Qua Glucagon-Like Peptide-1 Secretagogue via Intestinal Nutrient Sensors. Evid. Based Complement Alternat. Med. 2015, 9 171742 10.1155/2015/171742 PMC4693015 26788106

[29] Jadav P., Bahekar R., Shah S.R., Patel D., Joharapurkar A., Kshirsagar S. et al. (2012) Long-acting peptidomimetics based DPP-IV inhibitors. Bioorganic Med. Chem. Let 22, 3516–3521 10.1016/j.bmcl.2012.03.078 22503246

[30] Wedick N.M., Pan A., Cassidy A., Rimm E.B., Sampson L., Rosner B. et al. (2012) Dietary flavonoid intakes and risk of type 2 diabetes in US men and women. Am. J. Clin. Nutr. 95, 925–933 10.3945/ajcn.111.028894 22357723 PMC3302366

[31] Yaribeygi H., Atkin S.L. and Sahebkar A. (2019) Natural compounds with DPP-4 inhibitory effects: Implications for the treatment of diabetes. J. Cell. Biochem. 120, 1–5 10.1002/jcb.28467 30775811

[32] Huang P.-K., Lin S.-R., Chang C.-H., Tsai M.-J., Lee D.-N. and Weng C.-F. (2019) Natural phenolic compounds potentiate hypoglycemia via inhibition of Dipeptidyl peptidase IV. Sci. Rep. 9, 15585 10.1038/s41598-019-52088-7 31666589 PMC6821704

[33] Singh D.R., Arif T., Khan I. and Sharma D.P. (2014) Phytochemicals in antidiabetic drug discovery. J. Biomed. Therap. Sci. 1, 1–33

[34] Stohs S.J. and Bagchi D. (2015) Antioxidant, anti-inflammatory, and chemoprotective properties of Acacia catechu heartwood extracts. Phytother. Res. 29, 818–824 10.1002/ptr.5335 25802170 PMC6680240

[35] Ismail S. and Asad M. (2009) Immunomodulatory activity of *Acacia catechu*. Indian J. Physiol. Pharmacol. 53, 25–33 19810573

[36] Miraj S. and Kiani S. (2016) Pharmacological activities of *Carum carvi* L. Pharm Lett. 8, 135–138

[37] Veigas J.M., Narayan M.S., Laxman P.M. et al. (2007) Chemical nature stability and bioefficacies of anthocyanins from fruit peel of Syzygium cumini Skeels. Food Chem. 105, 619–627 10.1016/j.foodchem.2007.04.022

[38] de Albuquerque U., de Medeiros P., de Almeida A. et al. (2007) Medicinal plants of the caatinga (semi-arid) vegetation of NE Brazil: a quantitative approach. J. Ethnopharmacol. 114, 325–354 10.1016/j.jep.2007.08.017 17900836

[39] Goyal A., Sharma V., Upadhyay N. et al. (2014) Flax and flaxseed oil: an ancient medicine & modern functional food. J. Food Sci. Technol. 51, 1633–1653 25190822 10.1007/s13197-013-1247-9 PMC4152533

[40] Tolkachev O.N. and Zhuchenko A.A. (2000) Biologically active substances of flax: medicinal and nutritional properties (a review). Pharm. Chem. J. 34, 360–367 10.1023/A:1005217407453

[41] Bhowmik D., Biswas D. and Kumar K.P.S. (2011) Recent aspect of ethnobotanical application and medicinal properties of traditional Indian herbs *Santalum album*. Int. J. Chem. Res. 1, 21–27

[42] Hunt C.J. (2017) Cryopreservation: vitrification and controlled rate cooling. Methods Mol. Biol. 1590, 41–77 10.1007/978-1-4939-6921-0_5 28353262

[43] Singh S. and Singh R. (2012) Ethnomedicinal use of pteridophytes in reproductive health of tribal women of pachmarhi biosphere reserve, madhya pradesh, India. IJPSR 3, 4780–4790

[44] Anilakumar K.R., Pal A., Khanum F. et al. (2010) Nutritional, medicinal and industrial uses of sesame (Sesamum indicum L.) seeds - an overview. Agric. Conspec. Sci. 75, 159–168

[45] Hsu D., Chen S.J., Chu P.Y. et al. (2013) Therapeutic effects of sesame oil on monosodium urate crystal-induced acute inflammatory response in rats. Springer plus 2, 659 10.1186/2193-1801-2-659 24353977 PMC3866373

[46] Bektasoglu K.P. (2009) *Tamarindus indica* and its health related effects. Asian Pac. J. Trop Biomed. 4, 676–681

[47] Bachaya H.A., Iqbal Z., Khan M.N. et al. (2009) *In vitro* and *in vivo* anthelmintic activity of *Terminalia arjuna* bark. Int. J. Agric. Biol. 11, 273–278

[48] Dwivedi S. and Chopra D. (2014) Revisiting *terminalia arjuna*-an ancient cardiovascular drug. J. Tradit. Complement Med. 4, 224–231 10.4103/2225-4110.139103 25379463 PMC4220499

[49] Kumar V.S. and Navaratnam V. (2013) Neem (Azadirachta indica): Prehistory to contemporary medicinal uses to humankind. Asian Pac. J. Trop Biomed. 3, 505–514 10.1016/S2221-1691(13)60105-7 23835719 PMC3695574

[50] Ramachandran S., Faisal T.K., Anjumary J. et al. (2017) Comparative evaluation of hypoglycemic and hypolipidemic activity of various extract of *Anogeissus latifolia* bark in streptozotocin-induced diabetic rats. J. Complement Integr. Med. 14, 20160130 10.1515/jcim-2016-0130 28889731

[51] Tripathi R.M., Sen P.C. and Das P. (1979) Studies on the mechanism of action of Albizzia lebbeck, an Indian indigenous drug used in the treatment of atopic allergy. J. Ethnopharmacol. 1, 385–396 10.1016/S0378-8741(79)80003-3 544953

[52] Babu N.P., Pandikumar P. and Ignacimuthu S. (2009) Anti-inflammatory activity of *Albizia lebbeck* Benth., an ethnomedicinal plant, in acute and chronic animal models of inflammation. J. Ethnopharmacol. 125, 356–360 10.1016/j.jep.2009.02.041 19643557

[53] Fukai T., Oku Y., Hou A.J. et al. (2004) Antimicrobial activity of hydrophobic xanthones from Cudrania cochinchinensis against Bacillus subtilis and methicillin-resistant Staphylococcus aureus. Chem. Biodivers. 1, 1385–1390 10.1002/cbdv.200490101 17191916

[54] Danish M., Singh P., Mishra G. et al. (2011) *Cassia fistula* Linn. (Amulthus) - An important medicinal plant: A review of its traditional uses, phytochemistry and pharmacological properties. J. Nat. Prod. Plant Resour 1, 101–118

[55] Alam M.M., Siddiqui M.B. and Hussian W. (1990) Treatment of diabetes through herbal drugs in rural India. Fitoterapia 61, 240–242

[56] Mannan M.A., Khatun A. and Khan M.F.H. (2017) Antinociceptive effect of methanol extract of *Dalbergia sissoo* leaves in mice. BMC Complement. Altern. Med. 17, 1–13 10.1186/s12906-017-1565-y 28114964 PMC5260076

[57] Bhatt A., Rawal R.S. and Dhar U. (2006) Ecological features of a critically rare medicinal plant, *Swertia chirayita*, in Himalaya. Plant Species Biol. 21, 49–52 10.1111/j.1442-1984.2006.00150.x

[58] Kumar V. and Van Staden J. (2016) A review of *Swertia chirayita* (Gentianaceae) as a traditional medicinal plant. Front. Pharmacol. 1–14 26793105 10.3389/fphar.2015.00308 PMC4709473

[59] Ojha S., Alkaabi J., Amir N. et al. (2014) *Withania coagulans* fruit extract reduces oxidative stress and inflammation in kidneys of streptozotocin-induced diabetic rats. Oxid. Med. Cell Longev. 1–9 201436 10.1155/2014/201436 PMC4177777 25295146

[60] Sawant B.S., Alawe J.R. and Rasal K.V. (2016) Pharmacognostic study of *Glycyrrhiza glabra* Linn- a review. Inter. Ayurv Med. J. 4, 3188–3193

[61] Yibchok-Anun S., Adisakwattana S., Yao C.Y. et al. (2006) Slow acting protein extract from fruit pulp of Momordica charantia with insulin secretagogue and insulinomimetic activities. Biol. Pharm. Bull. 29, 1126–1131 10.1248/bpb.29.1126 16755004

[62] Abascal K. and Yarnell E. (2005) Using bitter melon to treat diabetes. Altern Complement Ther. 11, 179–184 10.1089/act.2005.11.179

[63] Parvez G.M.M. (2016) Pharmacological activities of mango (I): A review. J. Pharmacogn Phytochem. 5, 01–07

[64] Wauthoz N., Balde A., Balde E.S.D. et al. (2007) Ethnopharmacology of *Mangifera indica* L. bark and pharmacological studies of its main C-glucosylxanthone, mangiferin. Int. J. Biomed. Pharmaceut Sci. 1, 112–119

[65] Baliga M., Thilakchand K., Rai M. et al. (2012) *Aegle marmelos* (L.) Correa (Bael) and Its phytochemicals in the reatment and prevention of cancer. Integr. Cancer Ther. 12, 187–196 10.1177/1534735412451320 23089553

[66] Al-Oqail M.M., Farshori N.N., Al-Sheddi E.S. et al. (2013) *In vitro* cytotoxic activity of seed oil of fenugreek against various cancer cell lines. Asian Pacific J. Cancer Prev. 14, 1829–1832 10.7314/APJCP.2013.14.3.1829 23679282

[67] Nagulapalli Venkata K.C., Swaroop A., Bagchi D. et al. (2017) A small plant with big benefits: Fenugreek (*Trigonella foenum-graecum* Linn.) for disease prevention and health promotion. Mol. Nutr. Food Res. 61, 1–26 10.1002/mnfr.201600950 28266134

[68] Ikarashi N., Toda T., Okaniwa T., Ito K., Ochiai W. and Sugiyama K. (2011) Anti-obesity and anti-diabetic effects of Acacia polyphenol in obese diabetic KKAy mice fed high-fat diet. Evid Based Complement Alternat Med. 2011, 952031 10.1093/ecam/nep241 21799697 PMC3137845

[69] Giancarlo S., Rosa L.M., Nadjafi F. and Francesco M. (2006) Hypoglycaemic activity of two spices extracts: *Rhus coriaria* L. and *Bunium persicum* Boiss. Natur. Prod. Res. 20, 882–886 10.1080/14786410500520186 16753927

[70] Jana K., Bera T.K. and Ghosh D. (2015) Antidiabetic effects of *Eugenia jambolana* in the streptozotocin-induced diabetic male albino rat. Biomarkers Genom Med. 7, 116–124 10.1016/j.bgm.2015.08.001

[71] Governa P., Baini G., Borgonetti V., Cettolin G., Giachetti D., Magnano A.R. et al. (2018) Phytotherapy in the Management of Diabetes: A Review. Mol. 23, E105 10.3390/molecules23010105 PMC6017385 29300317

[72] Mani U.V., Mani I., Biswas M. and Kumar S.N. (2011) An open-label study on the effect of flax seed powder (*Linum usitatissimum*) supplementation in the management of diabetes mellitus. J. Diet. Supp. 8, 257–265 10.3109/19390211.2011.593615 22432725

[73] Kulkarni C.R., Joglekar M.M., Patil S.B. and Arvindekar A.U. (2012) Antihyperglycemic and antihyperlipidemic effect of *Santalum album* in streptozotocin induced diabetic rats. Pharmaceut Biol. 50, 360–365 10.3109/13880209.2011.604677 22129314

[74] Singh J.K., XXXX K.R., Obaidullah M. and Jha A.M. (2014) Effect of *Selaginella bryopteris* on diabetic swiss albino mice caused by alloxan. Int. J. Basic Applied Sci. Res. 1, 22–27

[75] Bhuvaneswari P. and Krishnakumari S. (2011) Antihyperglycemic potential of *Sesamum indicum* (linn) seeds in streptozotocin induced diabetic rats. Int. J. Pharm. Pharmaceut Sci. 4, 527–531

[76] Maiti R., Jana D., Das U. and Ghosh D. (2004) Antidiabetic effect of aqueous extract of seed of *Tamarindus indica* in streptozotocin-induced diabetic rats. J. Ethnopharmacol. 92, 85–91 10.1016/j.jep.2004.02.002 15099853

[77] Morshed M., Haque A., Rokeya B. and Ali L. (2011) Anti­hyperglycemic and lipid lowering effect of *Terminalia arjuna* bark extract on streptozotocin indiced type 2 diabetic model rats. Int. J. Pharm. Pharmaceut Sci. 3, 450–454

[78] Kasabri V., Flatt P.R. and Abdel-Wahab Y.H. (2010) *Terminalia bellirica* stimulates the secretion and action of insulin and inhibits starch digestion and protein glycation *in vitro*. British J. Nutr. 103, 212–217 10.1017/S0007114509991577 19723351

[79] Sunarwidhi A.L., Sudarsono S. and Nugroho A.E. (2014) Hypoglycemic effect of combination of *Azadirachta indica* A. Juss. and *Gynura procumbens* (Lour.) Merr. ethanolic extracts standardized by rutin and quercetin in alloxan-induced hyperglycemic rats. Adv. Pharmaceut Bult 4, 613–618 10.5681/apb.2014.090 PMC4312413 25671197

[80] Bhat M., Kothiwale S.K., Tirmale A.R., Bhargava S.Y. and Joshi B.N. (2011) Antidiabetic properties of *Azardiracta indica* and *Bougainvillea spectabilis*: *In vivo* studies in Murine diabetes model. Evid. Based Complement Alternat. Med. 2011, 561625 10.1093/ecam/nep033 19389871 PMC3136679

[81] Ramachandran S., Naveen K.R., Rajinikanth B., Akbar M. and Rajasekaran A. (2012) Antidiabetic, antihyperlipidemic and *in vivo* antioxidant potential of aqueous extract of *Anogeissus latifolia* bark in type 2 diabetic rats. Asian Pac. J. Trop Dis. 2, S596–S602 10.1016/S2222-1808(12)60229-1

[82] Patel P.A., Parikh M.P., Johari S. and Gandhi T.R. (2015) Antihyperglycemic activity of *Albizzia lebbeck* bark extract in streptozotocin-nicotinamide induced type II diabetes mellitus rats. Ayu 36, 335–340 27313423 10.4103/0974-8520.182752 PMC4895763

[83] Ahmed D., Kumar V., Verma A., Gupta P.S., Kumar H., Dhingra V. et al. (2014) Antidiabetic, renal/hepatic/pancreas/cardiac protective and antioxidant potential of methanol/dichloromethane extract of *Albizzia Lebbeck* Benth. stem bark (ALEx) on streptozotocin induced diabetic rats. BMC Complement Alt Med. 14, 243–243 10.1186/1472-6882-14-243 PMC4223618 25026962

[84] Antu K.A., Riya M.P., Mishra A., Anilkumar K.S., Chandrakanth C.K., Tamrakar A.K. et al. (2014) Antidiabetic property of *Symplocos cochinchinensis* is mediated by inhibition of alpha glucosidase and enhanced insulin sensitivity. PLoS ONE 9, e105829 10.1371/journal.pone.0105829 25184241 PMC4153544

[85] Einstein J.W., Mohd Rais M. and Mohd M.A. (2013) Comparative evaluation of the antidiabetic effects of different parts of *Cassia fistula* Linn, a Southeast Asian Plant. J. Chem. 2013, 10 10.1155/2013/714063

[86] Jarald E.E., Joshi S.B., Jain D.C. and Edwin S. (2013) Biochemical evaluation of the hypoglycemic effects of extract and fraction of *Cassia fistula* Linn. in alloxan-induced diabetic rats. Indian J. Pharmaceut. Sci. 75, 427–434 10.4103/0250-474X.119823 PMC3831724 24302797

[87] Pund K.V., Vyawahare N.S., Gadakh R.T. and Murkute V.K. (2012) Antidiabetic evaluation of *Dalbergia Sissoo* against alloxan induced diabetes mellitus in wistar albino rats. J. Nat. Prod. Plant Resour. 2, 81–88

[88] Niranjan P., Singh D., Prajapati K. and Jain S. (2010) Antidiabetic activity of ethanolic extract of *Dalbergia Sissoo* L. leaves in alloxan­induced diabetic rats. Int. J. Curr. Pharmaceut Res. 2, 24–27

[89] Thomson H., Ojo O., Flatt P. and Abdel-Wahab Y. (2014) Antidiabetic actions of aqueous bark extract of *Swertia chirayita* on insulin secretion, cellular glucose uptake and protein glycation. J. Exp. Integrat. Med. 4, 268–272 10.5455/jeim.160814.or.110

[90] Jaiswal D., Rai P.K. and Watal G. (2009) Antidiabetic effect of *withania coagulans* in experimental rats. Indian J. Clin. Biochem. 24, 88–93 10.1007/s12291-009-0015-0 23105813 PMC3453475

[91] Shukla K., Dikshit P., Shukla R. and Gambhir J.K. (2012) The aqueous extract of *Withania coagulans* fruit partially reverses nicotinamide/streptozotocin-induced diabetes mellitus in rats. J. Med. Food 15, 718–725 10.1089/jmf.2011.1829 22846078 PMC3407382

[92] Sil R., Ray D. and Chakraborti A.S. (2013) Glycyrrhizin ameliorates insulin resistance, hyperglycemia, dyslipidemia and oxidative stress in fructose-induced metabolic syndrome-X in rat model. Indian J. Exp. Biol. 51, 129–138 23923606

[93] Namazi N., Alizadeh M., Mirtaheri E. and Farajnia S. (2017) The effect of dried *Glycyrrhiza glabra* L. extract on obesity management with regard to PPAR-γ2 (Pro12Ala) Gene polymorphism in obese subjects following an energy restricted diet. Adv. Pharmaceut Bult 7, 221–228 10.15171/apb.2017.027 PMC5527236 28761824

[94] Ma C., Yu H., Xiao Y. and Wang H. (2017) *Momordica charantia* extracts ameliorate insulin resistance by regulating the expression of SOCS-3 and JNK in type 2 diabetes mellitus rats. Pharmaceut Biol. 55, 2170–2177 10.1080/13880209.2017.1396350 PMC6130557 29110587

[95] Joseph B. and Jini D. (2013) Antidiabetic effects of *Momordica charantia* (bitter melon) and its medicinal potency. Asian Pac. J. Trop Dis. 3, 93–102 10.1016/S2222-1808(13)60052-3

[96] Irondi E.A., Oboh G. and Akindahunsi A.A. (2016) Antidiabetic effects of *Mangifera indica* Kernel Flour-supplemented diet in streptozotocin-induced type 2 diabetes in rats. Food Sci. Nutr. 4, 828–839 10.1002/fsn3.348 27826432 PMC5090646

[97] Bhowmik A., Khan L.A., Akhter M. and Rokeya B. (2009) Studies on the antidiabetic effects of *Mangifera indica* stem-barks and leaves on nondiabetic, type 1 and type 2 diabetic model rats. Bangladesh J. Pharmacol. 4, 5 10.3329/bjp.v4i2.2488

[98] Mohammadi A., Gholamhosseinian A. and Fallah H. (2016) *Trigonella foenum-graecum* water extract improves insulin sensitivity and stimulates PPAR and γ gene expression in high fructose-fed insulin-resistant rats. Adv. Biomed. Res. 5, 54 27110551 10.4103/2277-9175.178799 PMC4817393

